# Digital Engagement and Health Behaviors Among Village Health Volunteers in Southern Thailand: A Cross-Sectional Study

**DOI:** 10.3390/ijerph23050618

**Published:** 2026-05-07

**Authors:** Deeyana Binhayeekonoh, Pussadee Laor, Nutnaree Nimsiri, Sujittra Hinwiset, Safeena Tohranee, Rohmatul Fajriyah, Wanvisa Saisanan Na Ayudhaya

**Affiliations:** 1 Department of Community Public Health, School of Public Health, Walailak University, 222 Thaiburi Sub-District, Thasala District, Nakhon Si Thammarat 80160, Thailand; deeyana.bi@mail.wu.ac.th (D.B.); safeena.to@mail.wu.ac.th (S.T.); 2 Department of Environmental Health, School of Health Science, Mae Fah Luang University, 333 Thasud Sub-District, Mueang District, Chiang Rai 57100, Thailand; pussadee.lao@mfu.ac.th; 3 Department of Statistics, Universitas Islam Indonesia, Jl. Kaliurang km. 14, 5 Sleman, Yogyakarta 55584, Indonesia; rfajriyah@uii.ac.id; 4 Excellent Centre for Public Health Research—EC for PHR, School of Public Health, Walailak University, 222 Thaiburi Sub-District, Thasala District, Nakhon Si Thammarat 80160, Thailand

**Keywords:** digital literacy, health behaviors, village health volunteers

## Abstract

**Highlights:**

**Public health relevance—How does this work relate to a public health issue?**

- Village Health Volunteers (VHVs) are central to community-based health promotion and disease prevention in Thailand.
- As digital tools become a routine part of public health work, understanding how VHVs use these tools—and how this relates to their own health behaviors—is increasingly important.

**Public health significance—Why is this work of significance to public health?**

- This study shows that health behaviors among community health volunteers are linked more to context and everyday use of digital tools than to digital skills alone.
- The findings add evidence on how health behaviors operate within digitally supported community health systems.

**Public health implications—What are the key implications or messages for practitioners, policy makers and/or researchers in public health?**

- Digital health strategies for community health volunteers should focus on how tools are used in daily work, not just on increasing digital use or skills.
- Policymakers and practitioners can build on existing platforms to support both service delivery and volunteer well-being.

**Abstract:**

Village Health Volunteers (VHVs) play an important role in Thailand’s community-based public health system, yet limited evidence is available on how their digital engagement relates to their own health behaviors. This cross-sectional study examined associations between sociodemographic characteristics, digital use frequency, digital literacy, and health behavior scores among 426 VHVs in southern Thailand. Data were collected using an online questionnaire that included the Mobile Device Proficiency Questionnaire (MDPQ-16) and a 23-item health behavior measure based on the Thai 3A2S framework. The median health behavior score was 61.0 (IQR 13.25). After adjustment, rural residence was associated with higher health behavior scores (β = 1.97, *p* = 0.043), whereas frequent digital use was associated with lower scores than infrequent use (β = −2.72, *p* = 0.010). Digital literacy was not independently associated with health behavior scores. The final model explained 4% of the variance, suggesting that additional factors may influence VHVs’ health behaviors. Overall, the findings indicate that digital literacy alone may not explain differences in health behaviors and that context may also play a role. Future research should examine these relationships using broader and more context-sensitive measures.

## 1. Introduction

Digital technologies have transformed how health information and services are accessed and used, with important implications for health promotion and disease prevention worldwide [1]. Broader digital transformation in health systems has also been recognized as an important contributor to progress toward Sustainable Development Goal 3 (SDG 3) [2]. As smartphones, Internet access, and social media have become a part of everyday life, digital health literacy—the ability to search for, evaluate, and apply health information from digital sources—has gained recognition as a key determinant of health behavior. Previous studies have shown that higher digital health literacy is associated with greater engagement in health-promoting activities, such as physical activity and preventive behaviors, even after accounting for sociodemographic differences [3].

Despite increasing attention paid to digital health literacy, evidence from community-level health actors remains limited. Research from China has demonstrated that digital literacy influences both participation in and diversity of digital health behaviors among rural populations, highlighting its potential role in reducing rural–urban health disparities [4]. Other studies focusing on online health information environments suggest that stronger digital health literacy supports healthier online health behaviors [5], underscoring its growing relevance in the digital health era [6].

In Thailand, Village Health Volunteers (VHVs) constitute a cornerstone of the community health system, supporting health promotion, disease prevention, and health communication at the household and community levels [7]. While prior studies have focused primarily on general health literacy among VHVs, considerably less attention has been paid to their digital literacy and patterns of digital engagement. This gap is increasingly salient, as Community-based health workers are increasingly expected to use digital tools for health education, data collection and monitoring, communication, and service coordination as part of routine public health practice [8]. Understanding how these digital expectations intersect with VHVs’ own health-related behaviors is therefore essential for informing sustainable digital health strategies.

Globally, digital technologies have become integral to public health systems, particularly in community-based health promotion and primary care. Digital health literacy—the capacity to seek, evaluate, and apply health information from digital sources—has been widely recognized as a determinant of effective health engagement and health-related behaviors [9,10,11]. However, international evidence consistently demonstrates that digital health literacy and digital engagement are unevenly distributed and strongly shaped by age, education, access to infrastructure, and local context [12,13,14]. Studies from low- and middle-income countries further suggest that while mobile technologies can enhance task efficiency and service delivery among community health workers, frequent digital use alone does not necessarily translate into healthier behaviors, underscoring the importance of contextual and organizational factors [15,16,17,18,19].

Despite growing global interest, empirical evidence examining the relationships among digital engagement, digital literacy, and health behaviors among community health volunteers remains limited, particularly in Southeast Asia. Thailand’s village health volunteer system provides a unique context for exploring these relationships within a well-established national workforce, which has progressively integrated digital technologies into routine public health practices.

In Thailand’s community health information system, village health volunteers routinely use multiple digital platforms in complementary ways. Smart VHVs functions as a national reporting and task-management application that connects volunteers’ household visits and screening activities with subdistrict health-promoting hospitals and district health offices, enabling near real-time data submission, case flagging, and feedback on priority health issues. In parallel, widely used messaging and social media platforms such as LINE and Facebook are employed for rapid dissemination of health alerts, coordination of outreach activities, and informal peer consultation, creating feedback loops between frontline workers and supervising staff that can support local decision-making and adaptive responses to emerging community needs [8,20]. Within this digitally mediated ecosystem, patterns of platform use, information flow, and responsiveness may shape how VHVs adopt and sustain health-promoting behaviors in their own daily lives.

Therefore, this study aimed to examine the associations between sociodemographic characteristics, digital use frequency, digital literacy, and health behavior scores among village health volunteers in the upper-southern region of Thailand. Rather than conceptualizing digital engagement solely as frequency of technology use, this study adopts a broader perspective that emphasizes how digital tools are embedded within everyday workflows and communication structures. Drawing on digital health and implementation science perspectives, we hypothesize that more purposeful, work-related digital engagement—such as the use of Smart VHVs and LINE groups for reporting, coordination, and peer support—may facilitate healthier routines and self-management practices among VHVs themselves.

## 2. Materials and Methods

### 2.1. Study Design and Setting

This cross-sectional analytical study was conducted in southern Thailand. Ethical approval was obtained from the Human Research Ethics Committee of Walailak University (approval number: WU-EC-PU-1-255-68; approval period: 29 August 2025 to 28 August 2026). Data were collected using an internet-based self-administered questionnaire among village health volunteers (VHVs) residing in two provinces, Nakhon Si Thammarat and Surat Thani.

Eligible participants were VHVs who met the following criteria: (1) officially registered in the national health information system with at least one year of work experience and (2) ability to access and complete an online questionnaire using an internet-enabled device (e.g., smartphone or tablet).

Urban and rural residences were classified according to national administrative definitions based on population density. Urban areas were defined as municipal areas with a population of 10,000 or more, while rural areas included all non-municipal areas, including sub-district (tambon) administrative organizations.

### 2.2. Sample Size Determination and Sampling

The sample size was calculated using the formula described by Krejcie and Morgan (1970) [21] at a significance level of α = 0.05. Based on an estimated population of 84,459 village health volunteers (VHVs) in Nakhon Si Thammarat and Surat Thani provinces, assuming a population proportion of 0.5 and a margin of error of 0.05, the minimum required sample size was 383 participants. The sample size was increased by 10% to account for potential non-response and incomplete questionnaires, resulting in a final target sample of 426 participants.

A multi-stage sampling technique was employed to ensure geographic representativeness across the upper southern region of Thailand, which is characterized by substantial heterogeneity in terrain and settlement patterns. In the first stage, two provinces, Nakhon Si Thammarat and Surat Thani, were selected by simple random sampling without replacement. In the second stage, one urban district and one rural district were selected from each province to reflect variations in socioeconomic and environmental contexts. Thasala District in Nakhon Si Thammarat and Mueang Surat Thani Districts were classified as urban areas, while Cha-uat District in Nakhon Si Thammarat and Phunphin District in Surat Thani were classified as rural areas in accordance with national administrative definitions.

**Figure 1:**
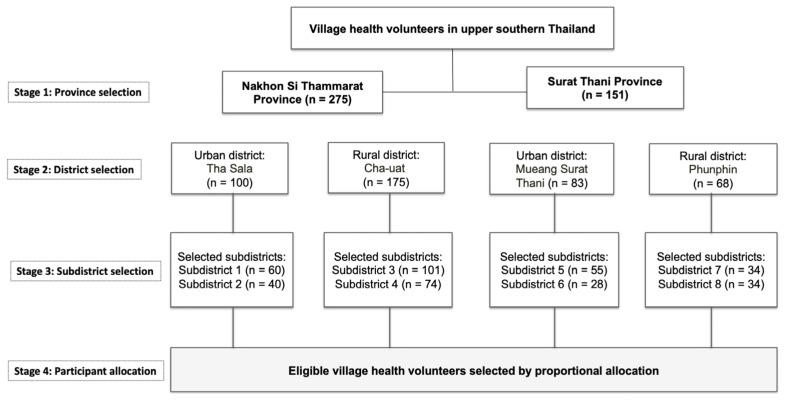
Flowchart of the multi-stage sampling process used to select village health volunteers in upper southern Thailand.

In the third stage, two sub-districts were randomly selected from each of the four districts, yielding a total of eight sub-districts. In the final stage, the number of VHVs recruited from each subdistrict was determined using proportional allocation based on the total number of registered volunteers in that subdistrict to ensure appropriate representation across all selected areas (Figure 1).

### 2.3. Data Collection and Measurements

#### 2.3.1. Data Collection

Data were collected using a self-administered online questionnaire among village health volunteers (VHVs) residing in the upper-southern region of Thailand. Community health centers and health-promoting hospital (HPH) officers facilitated participant recruitment by identifying eligible VHVs and disseminating the survey link. The participants completed the questionnaire via an online platform (Google Forms; Google LLC, Mountain View, CA, USA). Because the survey link was distributed through local coordinators, the final number of eligible VHVs who received or accessed the invitation could not be verified precisely; therefore, a reliable response rate could not be calculated.

The study questionnaire was developed by combining investigator-developed items on sociodemographic and digital characteristics with two structured measurement sections derived from existing instruments. The digital literacy section was adapted from the 16-item Mobile Device Proficiency Questionnaire (MDPQ-16), which was translated into Thai and contextually refined for use among Thai VHVs. The health behavior section was adapted from a 23-item instrument based on the Thai 3A2S framework. The content validity of all instruments was assessed by three experts, yielding item objective congruence (IOC) values ranging from 0.67 to 1.00. The overall reliability of the questionnaire was high (Cronbach’s alpha = 0.91).

#### 2.3.2. Research Measurements

The structured questionnaires consisted of three parts. An English-translated version of the questionnaire is provided as Table S1 in the Supplementary Materials. The main study variables were operationally defined as follows. Digital use frequency was assessed based on participants’ self-reported overall frequency of digital device use and categorized as either infrequent or frequent. Participants who reported “never,” “rarely,” or “sometimes” were classified as infrequent users, whereas those who reported “often” or “every day” were classified as frequent users. Digital literacy was defined as participants’ self-reported ability to perform mobile device-related tasks and was assessed using the 16-item Mobile Device Proficiency Questionnaire (MDPQ-16), with higher scores indicating greater digital literacy. Health behavior score was defined as the total score derived from the 23-item Thai 3A2S-based instrument, with higher scores indicating more health-promoting behaviors. General and work-related mobile application use was assessed separately as a multiple-response variable.

Part 1: Sociodemographic and digital characteristics. This section consisted of nine items on sex, age, educational attainment, monthly income, occupation, duration of work experience, frequency of digital device use, types of general applications used, and types of work-related applications used. Responses were collected using multiple-choice and fill-in-the-blank formats. Digital use frequency was assessed using a single item with five response categories: never, rarely (once a month or less), sometimes (once a week), often (3–6 times per week), and daily. For analysis, responses were dichotomized into infrequent use (never, rarely, or sometimes) and frequent use (often or daily). Participants were also asked to indicate all general applications (e.g., Facebook, LINE, TikTok, and YouTube) and work-related applications (e.g., Smart VHVs, VHVs Online, LINE, and Facebook) they used. Because participants could select more than one application, these data were treated as multiple-response items.

Part 2: Digital literacy. This section was adapted from the 16-item Mobile Device Proficiency Questionnaire (MDPQ-16) [22]. The instrument was translated into Thai and contextually refined to improve clarity and appropriateness for village health volunteers in Thailand. It evaluates proficiency across eight domains: mobile device basics, communication, data and file storage, internet use, calendar, entertainment, privacy, troubleshooting, and software management. Each domain included two items rated on a 5-point scale ranging from 1 (never tried) to 5 (very easily). Domain scores were averaged and summed to generate a total score ranging from 8.0 to 40.0, with higher scores indicating greater digital literacy. The instrument demonstrated excellent internal consistency (Cronbach’s α = 0.97). Participants with scores of 32 or higher were classified as having adequate digital literacy.

Part 3: Health Behaviors. This section was adapted from a 23-item instrument based on the Thai 3A2S framework [23]. The framework covers three health-promoting behaviors—nutrition, physical activity, and emotional management—and two risk-avoidance domains, namely smoking and alcohol consumption. It has been used in Thai health promotion practice as a practical model for encouraging healthy daily behaviors and risk avoidance. The 23 items were distributed across five domains: nutrition (8 items), physical activity (3 items), emotional management (6 items), smoking (3 items), and alcohol consumption (3 items). Responses were rated on a 5-point scale, and higher total scores indicated more health-promoting behaviors. The instrument demonstrated acceptable internal consistency in this study (Cronbach’s α = 0.85). Health behavior scores were analyzed as continuous variables in the main analyses.

### 2.4. Data Analysis

Data were analyzed using IBM SPSS Statistics, version 29.0 (IBM Corp., Armonk, NY, USA). Descriptive statistics were used to summarize the participant characteristics. Categorical variables were reported as frequencies and percentages, while continuous variables were summarized using medians and interquartile ranges owing to non-normal distributions. Bivariable analyses were conducted using independent *t*-tests and Spearman’s rank correlation coefficients as appropriate. Although several continuous variables were summarized using medians and interquartile ranges because of non-normality, independent *t*-tests were used for group comparisons of health behavior scores because the sample size was sufficiently large and the test is generally robust to moderate departures from normality. Multivariable linear regression was performed to identify factors independently associated with health behavior scores, with all variables entered simultaneously, based on theoretical relevance. Monthly income was rescaled in units of 1000 THB before inclusion in the regression model to improve the interpretability of the coefficient. The model assumptions were assessed and met. Statistical significance was set at *p* < 0.05.

## 3. Results

### 3.1. Sociodemographic, Digital, and Health Behavior Characteristics of Participants of VHVs

Table 1 presents the sociodemographic, digital, and health behavior characteristics of the 426 village health volunteers included in this study. Most participants were female (92.3%), and over half were aged 51 years or older (53.8%). More than half had completed secondary education or higher (54.9%), while the remainder had lower educational attainment. The majority were engaged in non-farm occupations (60.8%), whereas the rest reported farm-based work.

The median monthly income was 8000 THB (IQR = 5000), with incomes ranging widely from 1000 THB to 50,000 THB. The participants had substantial experience as village health volunteers, with a median service duration of 13 years (IQR = 15). More than half of the VHVs lived in urban areas (57.0%), whereas 43% resided in rural communities. Approximately two-thirds of the participants reported frequent use of digital technologies (66.9%). The median digital literacy score was 28.0 (IQR = 8.0), and the median health behavior score was 61.0 (IQR = 13.25).

**Figure 2:**
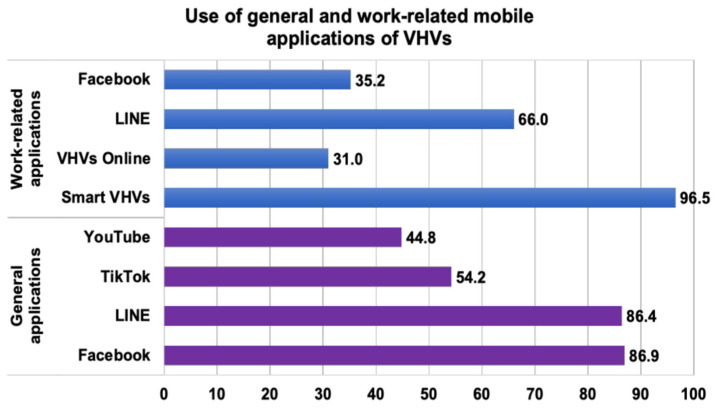
Distribution of general and work-related mobile application use among VHVs. Note: Participants could report more than one application; therefore, the percentages do not sum to 100%.

The patterns of mobile device application use are shown in Figure 2. Most VHVs used common communication and social media applications, particularly Facebook (86.9%) and LINE (86.4%). More than half of the participants reported using TikTok (54.2%) or YouTube (44.8%). With regard to work-related applications, nearly all the participants used the national smart VHVs application (96.5%). In addition, LINE (66%) and Facebook (35.2%) were commonly used for work-related communication, whereas fewer participants reported using VHVs Online (31%). Overall, these findings reflect a high level of engagement with both general and work-related mobile applications among VHVs, providing an important context for subsequent analyses of digital literacy and health behaviors.

### 3.2. Bivariable Associations Between Sociodemographic, Digital Factors, and Health Behavior Scores

Bivariate associations between sociodemographic characteristics, digital factors, and health behavior scores are summarized in Table 2. Health behavior scores did not differ significantly by sex (*p* = 0.384), age group (*p* = 0.086), or employment status (*p* = 0.162). In contrast, participants with lower educational attainment (<secondary school) reported significantly higher mean health behavior scores than those with secondary education or higher (*p* = 0.030). Area of residence was also associated with health behavior scores, with rural VHVs demonstrating higher scores than their urban counterparts (*p* = 0.021).

Digital engagement showed a distinct pattern. VHVs who reported infrequent digital use had significantly higher mean health behavior scores compared with those reporting frequent use (*p* < 0.001). Spearman’s correlation analyses indicated no significant associations between health behavior scores and monthly income (ρ = 0.022, *p* = 0.657) or duration of work as a village health volunteer (ρ = 0.062, *p* = 0.202). However, digital literacy scores demonstrated a weak but statistically significant negative correlation with health behavior scores (ρ = −0.127, *p* = 0.009). Variables demonstrating statistical significance or theoretical relevance were subsequently entered into multivariable regression analyses.

### 3.3. Factors Associated with Health Behavior Scores: Multivariable Linear Regression Analysis

Table 3 presents the results of the multivariable linear regression analysis. Overall, the model was statistically significant (F(9, 416) = 2.99, *p* = 0.002) and explained a modest proportion of the variance in health behavior scores (R^2^ = 0.061; adjusted R^2^ = 0.040). After adjustment for the variables included in the model, area of residence and digital use frequency remained significantly associated with health behavior scores. Rural VHVs had higher health behavior scores than urban VHVs (B = 1.97, 95% CI: 0.06–3.89; *p* = 0.043), while frequent digital use was associated with lower scores than infrequent use (B = −2.72, 95% CI: −4.79 to −0.65; *p* = 0.010). To reflect the relative contribution of each factor, standardized beta coefficients were also examined. Among the variables in the model, frequent digital use showed the largest standardized coefficient in relation to lower health behavior scores (standardized β = −0.13), followed by rural residence, which showed a smaller positive contribution (standardized β = 0.10). The remaining variables—sex, age group, education level, occupation, monthly income, duration of volunteer service, and digital literacy—were not significantly associated with health behavior scores after adjustment.

## 4. Discussion

This study suggests that health behaviors among village health volunteers (VHVs) are shaped more by local context than by digital literacy alone. After adjustment, the area of residence remained associated with health behavior scores, while digital literacy did not appear to have an independent association. In this study, digital engagement was assessed through digital use frequency and digital literacy, so the findings should be interpreted in relation to these measured variables rather than as evidence of broader causal mechanisms. Taken together, the results suggest that digital skills are part of a wider set of social, organizational, and environmental conditions that influence everyday health practices. This means that digital skills may be necessary, but on their own, may not be sufficient to support healthier behaviors. This interpretation is in line with previous studies showing that the relationship between digital literacy and health behavior depends on factors such as age, socioeconomic position, and access to digital infrastructure, rather than following a uniform or linear pattern [24,25]. Evidence from China further supports this view, showing that digital literacy may promote specific health behaviors, such as a healthier diet and physical activity, among rural older adults, while working differently across settings and population groups [4,26,27].

The adjusted R^2^ in this study was modest (0.040), suggesting that the variables included in the model explained only a limited part of VHVs’ health behaviors. Although modest explanatory power is common in behavioral research, in this study, it also suggests that important determinants of VHVs’ health behaviors were not captured in the model. Health behaviors are shaped by a range of psychological, social, cultural, and environmental influences that are often difficult to measure fully in cross-sectional surveys. In the context of a large community health volunteer workforce, even modest associations may still be meaningful, particularly when they relate to everyday routines and local working conditions [13,25]. At the same time, factors such as internet connectivity, device access, platform usability, information quality, workload, and organizational support may also influence health behaviors among VHVs and could help explain additional variation in future studies. In addition, the use of self-reported and dichotomized measures (e.g., digital use frequency) may have limited the sensitivity of the model to detect more nuanced patterns of digital engagement. These may include organizational support, workload, digital infrastructure, and integration of digital tools into routine tasks, which warrant further investigation.

International evidence consistently indicates that digital health literacy interacts with sociodemographic and contextual factors, rather than exerting a uniform influence on health behaviors. Studies from Korea highlight persistent inequalities in digital engagement, with lower digital health literacy among older adults associated with reduced mobile health use [28]. Cross-sectional evidence from rural China similarly reports that digital literacy positively influences both participation in and diversity of digital health behaviors, with health literacy amplifying the diversity of online health engagement [4]. Studies from Germany and the United Kingdom also demonstrate substantial variation in digital health literacy by age and social characteristics, with corresponding differences in health status and health-related behaviors [10]. A population-based study also demonstrated that digital health literacy is closely linked to online health behaviors shaped by exposure to Internet-based information and advertising, highlighting the role of literacy in navigating complex digital environments [6]. Consistent with this, a systematic review reported that higher digital health literacy was generally associated with improved health outcomes, although the strength and direction of the associations varied across contexts and health domains [29].

The observation that more frequent digital use did not necessarily correspond to healthier behaviors in our adjusted models is consistent with broader literature on digital interventions. This pattern suggests that simple exposure to digital technologies may be insufficient, on its own, to support healthier behaviors in everyday practice. Rather, the relevance of digital tools may depend on how they are incorporated into VHVs’ routine responsibilities, communication structures, and local support systems. Digital health literacy interventions can improve users’ health knowledge, but their effects on sustained behavior change are often modest in the absence of complementary support mechanisms or structured engagement [30]. Likewise, systematic reviews have reported only moderate correlations between eHealth literacy and health-related behaviors, indicating that factors beyond literacy itself—such as motivation, self-efficacy, and environmental support—also play important roles in behavior change [25].

Evidence from specific population groups further illustrates these dynamics. Studies among Chinese internet users have shown that age and education strongly shape digital health literacy, with older adults often reporting lower literacy levels that may constrain online health engagement [26]. Research from Sweden similarly indicates that adequate health literacy supports greater use of online health portals and electronic health records, highlighting the combined influence of digital and general health literacy on information-seeking behaviors [11]. Among older adults in Korea, higher digital literacy is associated with healthier lifestyle behaviors and better perceived health status, underscoring the relevance of digital engagement for health promotion in aging societies [29]. Large-scale survey data from Denmark further showed that higher digital health literacy was associated with increased physical activity and adherence to WHO recommendations, demonstrating positive behavioral associations at the population level [3]. These findings highlight that, across diverse settings, digital engagement and contextual readiness interact in complex ways to shape health behaviors [26].

In contrast to studies focusing on the general population or specific age groups, the present findings highlight the importance of purpose-driven digital engagement within a community health volunteer workforce. The routine use of familiar platforms such as LINE, Facebook, and the Smart VHVs application suggests that these technologies are embedded in daily communication, coordination, and task execution among Thai VHVs. This pattern supports evidence from other settings indicating that digital tools integrated into everyday professional roles are more likely to influence health-related behaviors than isolated digital competencies or stand-alone applications [8,31].

Overall, these international comparisons reinforce that digital literacy and engagement are context dependent. Institutional support, integration with routine tasks, and reliance on trusted platforms appear to strengthen the potential impact of digital engagement on health behaviors. Recent review evidence suggests that the effectiveness of digital health literacy interventions may depend in part on cultural tailoring, sustained support, and attention to broader social needs that influence access and engagement [32]. By highlighting the interplay between individual capacity, local context, and routine digital work practices, this study adds to the growing evidence on digitally supported community health systems, particularly in settings where digital tools are already embedded in routine public health work [20,32].

### 4.1. Practice Implications

The findings of this study suggest that digital engagement among VHVs should not be understood only in terms of digital literacy or frequency of use alone [18,19]. Rather, its relevance appears to depend on how digital tools are embedded in routine work practices, communication patterns, and local contexts [15,18,19]. In the present study, frequent digital use was not associated with better health behavior scores, whereas rural residence was positively associated with healthier behaviors. This pattern suggests that simply spending more time using digital tools may not be sufficient to support healthier behaviors, particularly when digital use is not closely aligned with practical needs, supportive environments, or meaningful health-related routines [15,18,19].

The observed patterns of mobile application use also indicate that digital technologies are already woven into everyday VHV practice [8,20]. The widespread use of Facebook and LINE suggests that familiar social platforms remain important for communication and information exchange, while the near-universal use of Smart VHVs reflects the integration of digital tools into routine public health activities rather than their use as separate add-ons [8,33]. Taken together, these findings suggest that digital health initiatives for VHVs may be more effective when they build on existing workflows and commonly used platforms, rather than relying on stand-alone technologies or generic increases in digital use [8,20,33].

These findings also have implications for how digital support for VHVs is conceptualized. Rather than treating digital engagement as an isolated individual attribute, it may be more useful to view it as part of a broader socio-technical environment in which communication channels, reporting systems, supervision, and local service demands interact [15,18,19]. From this perspective, the relationship between digital engagement and health behaviors is likely to be shaped not only by individual skills but also by how digital tools fit into the practical realities of community health work.

### 4.2. Implications for Thailand’s VHV System

In Thailand, the VHV system provides a particularly relevant setting for understanding how digital engagement is incorporated into community health work [7,8,33]. The routine use of Smart VHVs alongside widely used communication platforms such as LINE and Facebook suggests that digital tools are already embedded in established work processes rather than being introduced as separate add-ons [8,33]. This is important because it indicates that digital capacity in the VHV system may be strengthened not only through formal training, but also through repeated use of familiar tools in everyday communication, reporting, and coordination [34].

Building on these observations, a digitally enabled VHV system may be understood as involving four linked elements: clearly defined volunteer roles that combine community outreach with attention to volunteers’ own well-being; a core set of digital tools for communication, reporting, and coordination; structured information flows from households and communities into primary care systems, with timely feedback to volunteers; and measurable outcomes that reflect both community service and VHVs’ own health behaviors. In this view, digital engagement is not an end in itself, but a practical mechanism connecting information flow, service coordination, and behavior-related support across different levels of primary care [8,15,20].

Recent Thai evidence on VHV 4.0 competency development also points to increasing attention to communication, information use, and technology-supported service activities in volunteer practice [34]. This supports the relevance of a framework that places digital engagement within broader patterns of routine work, rather than treating it as a stand-alone technical skill [19,34]. At the same time, the present findings should be interpreted with appropriate caution because this study used a cross-sectional design and self-reported measures. It cannot be determined whether digitally integrated work routines directly improve health behaviors, and other unmeasured factors may also contribute to the observed associations. Nevertheless, the results suggest that future efforts to strengthen digital health systems for VHVs may benefit from focusing not only on digital capability, but also on how digital tools are incorporated into everyday practice in ways that are realistic, useful, and sustainable [8,15,19,34].

## 5. Strengths and Limitations

This study has several notable strengths. The relatively large sample of village health volunteers provided sufficient statistical power to examine associations between digital factors and health behavior scores across different community settings. The inclusion of both rural and urban participants also allowed meaningful contextual comparisons within the upper-southern region of Thailand. In addition, assessing both digital use frequency and digital literacy enabled a more nuanced analysis by distinguishing between digital engagement and digital skills. The use of multivariable linear regression with adjustment for key sociodemographic and work-related characteristics further strengthened the analysis, and the use of continuous health behavior scores increased sensitivity for detecting associations that might have been missed with categorical outcomes.

Several limitations should also be considered. First, the cross-sectional design precludes causal inference, and the temporal direction of the observed associations cannot be established. Second, because data were collected through self-reported online questionnaires, the findings may have been affected by recall bias or social desirability bias, and objective measures of digital engagement or health behaviors were not available. Third, participation required access to an internet-enabled device, which may have favored VHVs who were already more digitally connected and therefore introduced selection bias. Furthermore, the categorization of digital use frequency into two groups may have reduced variability and masked more detailed usage patterns, which could influence the observed associations. In addition, because the number of eligible VHVs who received or accessed the survey link could not be verified precisely, a reliable response rate could not be calculated. Fourth, although the model identified statistically significant associations, the explained variance was modest, indicating that health behaviors are shaped by multiple factors beyond those captured in this study. This modest explanatory power suggests that important behavioral and system-level determinants were not included in the model. More detailed indicators of the digital and social environment, such as internet connectivity, device access, information quality, workload, platform usability, and organizational support, were not assessed and should be considered in future research. Finally, because the sample was drawn from selected districts in two provinces in southern Thailand, the findings may not be fully generalizable to all VHVs nationwide or to other community health systems.

## 6. Conclusions

This study suggests that health behaviors among village health volunteers in southern Thailand are shaped by the interaction between digital engagement and local context, rather than by digital literacy or frequency of technology use alone. More frequent digital use was not associated with better health behaviors, underscoring that how digital tools are integrated into everyday routines may be more important than how often they are used.

The Thai VHV system offers a useful foundation for integrating digital functions into routine public health work by building on established roles and familiar platforms. Rather than focusing only on increasing digital activity, future initiatives may be more effective if they embed behavior-supportive prompts, simple feedback mechanisms, and tailored messages within existing systems such as Smart VHVs. Designing digital features that align with VHVs’ everyday workflows may help improve both service delivery and the health of the volunteer workforce. Future research should incorporate broader contextual and system-level variables to better explain variation in health behaviors.

## Figures and Tables

**Table 1:** Sociodemographic, digital, and health behavior characteristics of VHVs (*n* = 426).

Characteristics	*n* (%) or Median (IQR)
Sex	
Male	33 (7.7)
Female	393 (92.3)
Age (years)	
26–50	197 (46.2)
≥51	229 (53.8)
Education level	
<Secondary school	192 (45.1)
≥Secondary school	234 (54.9)
Employment	
Non-farm	259 (60.8)
Farm-based	167 (39.2)
Monthly income (THB)	8000 (IQR 5000)
Min–Max	1000–50,000
VHVs Duration (Years)	13.0 (IQR 15.0)
Min–Max	1–49
Area of residence	
Rural	183 (43.0)
Urban	243 (57.0)
Digital Use Frequency	
Infrequent	141 (33.1)
Frequent	285 (66.9)
Digital literacy score	28.00 (IQR 8.0)
Min–Max	11.5–40.0
Health behavior score	61.00 (IQR 13.25)
Min–Max	37.0–95.0

Note: Values are presented as numbers (percentage) for categorical variables and medians (interquartile range) for continuous variables due to non-normal distributions.

**Table 2:** Unadjusted associations between sociodemographic and digital factors and health behavior scores among village health volunteers (n = 426).

Variables	Health Behavior Score	Statistical Test	*p*-Value
Sex		*t*-test	0.384
Male	63.52 (10.66)		
Female	61.96 (9.81)		
Age (years)		*t*-test	0.086
26–50	61.19 (9.95)		
≥51	62.83 (9.76)		
Education level		*t*-test	0.030 *
<Secondary school	63.22 (9.89)		
≥Secondary school	61.14 (9.77)		
Employment		*t*-test	0.162
Non-farm	61.54 (9.91)		
Farm-based	62.91 (9.78)		
Area of residence		*t*-test	0.021 *
Rural	63.04 (9.66)		
Urban	60.80 (10.02)		
Digital Use Frequency		*t*-test	<0.001 **
Infrequent	64.38 (10.84)		
Frequent	60.93 (9.16)		
Monthly income (THB)	0.022	Spearman	0.657
VHVs Duration (Years)	0.062	Spearman	0.202
Digital literacy score	−0.127	Spearman	0.009 *

Note: ** *p* < 0.001, * *p* < 0.05; Values for categorical variables are presented as mean (SD) health behavior scores and compared using independent *t*-tests. For continuous variables, Spearman’s rank correlation coefficients are shown because of non-normal distributions.

**Table 3:** Multivariable linear regression analysis of factors associated with health behavior scores among village health volunteers (n = 426).

Variable	B (Unstandardized)	95% CI for B	Standardized β	*p*-Value
Sex (female vs male)	–1.47	–4.97, 2.03	–0.04	0.409
Age group (≥51 vs. 26–50 years)	0.34	–1.90, 2.57	0.02	0.767
Education level (≥secondary vs. <secondary)	–1.15	–3.19, 0.89	–0.06	0.267
Occupation (farm-based vs. non-farm)	1.68	–0.27, 3.63	0.08	0.091
Area of residence (rural vs. urban)	1.97	0.06, 3.89	0.10	0.043 *
Digital use frequency (frequent vs. infrequent)	−2.72	–4.79, −0.65	−0.13	0.010 *
Monthly income (per 1000 THB)	0.16	−0.06, 0.29	0.07	0.186
VHVs duration (years)	0.01	–0.10, 0.12	0.01	0.857
Digital literacy score	−0.12	–0.29, 0.06	−0.07	0.201

Note: * *p* < 0.05; Model fit: R^2^ = 0.061; Adjusted R^2^ = 0.040; F(9, 416) = 2.99; *p* = 0.002. Standardized β coefficients are presented to indicate the relative contribution of each factor in the model. All variables were entered simultaneously into the model. Linear regression assumptions were assessed and met. Variance inflation factors (VIFs) were below 2, indicating no multicollinearity.

## Data Availability

The raw data supporting the conclusions of this article will be made available by the authors on request.

## Supplementary Materials

The following supporting information can be downloaded at: https://www.mdpi.com/article/10.3390/ijerph23050618/s1, Table S1: Questionnaire.
