# Supplementary material for: Digital Engagement and Health Behaviors Among Village Health Volunteers in Southern Thailand: A Cross-Sectional Study
Source: Int J Environ Res Public Health. 2026 May 7;23(5):618. doi: 10.3390/ijerph23050618 (PMC13206079; doi:10.3390/ijerph23050618)
Supplement: Supplementary file 1 [file ijerph-23-00618-s001.zip › ijerph-4162316-supplementary.pdf]

Table S1. English-Translated version of the questionnaire used in this study.

Note: The original questionnaire was administered in Thai. The questionnaire comprised three parts: (1) sociodemographic and digital characteristics, (2) digital literacy assessed using the 16-item Mobile Device Proficiency Questionnaire (MDPQ-16), and (3) health behaviors assessed using a 23-item instrument based on the Thai 3A2S framework. The translated version is provided to facilitate review and interpretation by international readers.

Part 1. Sociodemographic and Digital Characteristics (9 Items)

Instructions: Please place a check mark (✓) in the box that best matches your actual circumstances. Please answer all questions truthfully.

1. **Sex**
   ☐ Male
   ☐ Female
2. **Age** ________ years
3. **Educational Attainment**
   ☐ Lower than upper secondary school
   ☐ Upper secondary school or vocational certificate
   ☐ Bachelor's degree, high vocational certificate or diploma
   ☐ Higher than bachelor's degree
4. **Monthly Income** ________ baht/month
5. **Occupation**
   ☐ Unemployed
   ☐ Merchant / business owner
   ☐ Farmer / agriculturist
   ☐ Company employee / factory worker
   ☐ General hired worker / laborer
   ☐ Other (please specify) _________
6. **Duration of Work Experience as a Village Health Volunteer** ________ years
7. **Frequency of Digital Device Usage**
   ☐ Never
   ☐ Rarely (once a month or less)
   ☐ Sometimes (once a week)
   ☐ Often (3-6 times a week)
   ☐ Daily
8. **General Applications Used**

☐ Facebook
☐ LINE
☐ TikTok
☐ YouTube
☐ Other (please specify) ________

9. **Work-related Applications Used**
☐ Smart VHV (Smart Or Sor Mor)
☐ VHV Online (Or Sor Mor Online)
☐ LINE
☐ Facebook
☐ Other
(please specify) ________

Part 2: Digital literacy questionnaire for Village Health Volunteers (16-item).

Instructions: Please place a check mark (✓) in the box that best describes your ability. Please assess how easily you can perform each task using a mobile device such as a smartphone or tablet.

No.	Using your mobile device, you can:	Never tried	Not at all	Not very easily	Somewhat easily	Very easily
1.	**Mobile Device Basics**					
	1.1 Navigate on-screen menus using the touchscreen					
	1.2 Use the on-screen keyboard to type					
2.	**Communication**					
	2.1 Send emails					
	2.2 Send pictures by email					
3.	**Data and File Storage**					
	3.1 Transfer information (files such as music, pictures, documents) on my mobile device to my computer					
	3.2 Transfer information (files such as music, pictures, documents) on my computer to my mobile device					
4.	**Internet Use**					
	4.1 Find information about my hobbies and interests on the Internet					
	4.2 Find health information on the Internet					
5.	**Calendar**					

No.	Using your mobile device, you can:	Never tried	Not at all	Not very easily	Somewhat easily	Very easily
	5.1 Enter events and appointments into a calendar					
	5.2 Check the date and time of upcoming and prior appointments					
**6.**	**Entertainment**					
	6.1 Use the device's online store (e.g., Apple App Store or Google Play Store) to find games or other forms of entertainment					
	6.2 Listen to music					
**7.**	**Privacy**					
	7.1 Set up a password to lock or unlock the device					
	7.2 Erase Internet browsing history and temporary files					
**8.**	**Troubleshooting and Software Management**					
	8.1 Update games and other applications					
	8.2 Delete games and other applications					

Note: *The following scale applies to all items in this section: Never tried (1), Not at all (2), Not very easily (3), somewhat easily (4), very easily (5).*

Part 3: Health Behaviors Based on the Thai "3A2S" framework

3.1 Dietary, Physical Activity, and Emotional Management Behaviors
Instructions: Please place a check mark (✓) in the box that best describes your behavior during the past week.

Question	Average frequency of practice in 1 week				
	Every day	5-6 days	3-4 days	1-2 days	Never
**1. Dietary Behavior**					
1) How often do you eat cooked and hygienic food?					
2) How often do you eat a varied diet that includes all five food groups in appropriate proportions within a meal?					
3) How often do you consume at least five servings (approximately 400 g) of fruits and vegetables combined per day?					
4) How often do you consume sweet snacks (e.g., cakes, chocolate, or ice cream) or sweetened beverages?					
5) How often do you consume high-fat foods (e.g., pork leg with rice, chicken rice, or coconut milk curries)?					
6) How often do you consume salty or high-sodium foods (e.g., sausages, crispy snacks)?					
7) How often do you consume processed, fried, grilled/smoked, or artificially colored foods?					
8) How often do you drink 6-8 glasses of clean water per day?					
**2. Physical Activity Behaviors**					
1) How often do you stand up or move your body every 2 hours during the day?					

Question	Average frequency of practice in 1 week				
	Every day	5-6 days	3-4 days	1-2 days	Never
2) How often do you run, play, or exercise until your heart beats faster or you breathe harder than usual, accumulating at least 1 hour per day?					
3) How often do you practice muscle-strengthening exercises (e.g., push-ups, wall push-ups, sit-ups)?					
**3. Emotional Management Behaviors**					
1) How often do you observe your own emotions or feelings each day?					
2) How often do you use relaxation methods when feeling stressed or unwell (e.g., playing sports, listening to music, watching movies)?					
3) How often do you engage in activities that keep your mind cheerful (e.g., talking with friends, hobbies, raising pets, singing)?					
4) How often can you allocate sufficient time for your personal life and family?					
5) How often do you sleep 9–10 hours per day?					
6) How often are you able to avoid situations that cause stress (e.g., arguments or conflict)?					

3.2 Smoking Behaviors

1) Do you currently smoke cigarettes or e-cigarettes?

☐ Smoker

☐ Non-smoker

2) During the past week, how often were you near or in a group with people smoking cigarettes or e-cigarettes?

☐ Every day
☐ 5–6 days
☐ 3–4 days
☐ 1–2 days
☐ Never

3) How often do you refuse when invited to smoke cigarettes or e-cigarettes by friends, family, or close acquaintances?

☐ Every time or never invited
☐ Often
☐ Sometimes
☐ Rarely
☐ Never

3.3 Alcohol Consumption Behaviors

1) During the past month, how often did you drink alcohol or alcoholic beverages?

☐ 4 times or more per week
☐ 2–3 times per week
☐ 2–4 times per month
☐ 1–2 days per month
☐ Non-drinker

2) During the past month, how often were you near or in a group with people drinking alcohol?

☐ 4 times or more per week
☐ 2–3 times per week
☐ 2-4 times per month
☐ 1–2 days per month
☐ Never

3) How often do you refuse when invited to drink alcohol by friends, family, or close acquaintances?

☐ Every time or never invited
☐ Often
☐ Sometimes
☐ Rarely
☐ Never
